# Chronic Intake of Micrograms of Abscisic Acid Improves Glycemia and Lipidemia in a Human Study and in High-Glucose Fed Mice

**DOI:** 10.3390/nu10101495

**Published:** 2018-10-12

**Authors:** Mirko Magnone, Giovanna Leoncini, Tiziana Vigliarolo, Laura Emionite, Laura Sturla, Elena Zocchi, Giovanni Murialdo

**Affiliations:** 1 Department of Experimental Medicine (DIMES), University of Genova, Viale Benedetto XV 1, 16132 Genova, Italy; detiviglia@libero.it (T.V.); laurasturla@unige.it (L.S.); 2 Nutravis S.r.l., Via Corsica 2/9, 16128 Genova, Italy; 3 Department of Internal Medicine, IRCCS Ospedale San Martino, Largo R. Benzi 1, 16132 Genova, Italy; giovanna.leoncini@unige.it (G.L.); gmurialdo@unige.it (G.M.); 4 Animal Facility, IRCCS Ospedale San Martino, Largo R. Benzi 1, 16132 Genova, Italy; laura.emionite@hsanmartino.it

**Keywords:** prediabetes, metabolic syndrome, food supplement, glucose tolerance, cardiovascular risk

## Abstract

We tested the effect of chronic low-dose abscisic acid (ABA), a phytohormone-regulating human glucose tolerance, on the metabolic parameters that are dysregulated in prediabetes and metabolic syndrome (MS).Ten healthy subjects received 1 µg ABA/Kg body weight (BW)/day as an ABA-rich food supplement: (i) the glycemia profile after a carbohydrate-rich meal, with or without supplement, was compared; (ii) fasting blood glucose (FBG), glycated hemoglobin (HbA1c), total cholesterol (TC), and body mass index (BMI) after 75 days of daily supplementation of a habitual Mediterranean diet were compared with starting values.CD1 mice were fed a high-glucose diet with or without synthetic ABA (1 µg/Kg BW) for 4 months and the same parameters investigated in the human study were compared. The food supplement significantly reduced the area under the curve of glycemia after a carbohydrate-rich meal and FBG, HbA1c, TC, and BMI after chronic treatment. ABA-treated mice showed a significant reduction of HbA1c, TC, and body weight gain compared with untreated controls. The combined results from the human and murine studies allow us to conclude that the observed improvement of the metabolic parameters can be attributed to ABA and to advocate the use of ABA-containing food supplements in prediabetes and/or MS.

## 1. Introduction

Type 2 diabetes mellitus (T2D) has reached epidemic proportions worldwide. It has been estimated that, globally, over 8% of adults, corresponding to more than 380 million people, have T2D and this number is set to rise beyond 600 million in less than 25 years [1]. Furthermore, the prevalence of insulin resistance, the cause of prediabetes and an independent risk factor for cardiovascular disease and metabolic syndrome (MS), is even more widespread [2,3,4]. To date, the number of prediabetic subjects is estimated at 30 million in Europe and at 86 million in the United States (i.e., approximately 35% of the U.S. population). While T2D is irreversible, prediabetes, the most important risk factor to develop T2D, can be reverted to a healthy metabolic condition through lifestyle interventions, which require a lifelong commitment, and suffer low compliance.

While the appropriateness and cost-effectiveness of pharmacological interventions in prediabetic subjects are being debated, nutraceuticals and food supplements are attracting attention as a viable strategy to address the need for safe, lifelong interventions to reduce the risk of developing diabetes and dyslipidemia. Botanicals have been extensively used in traditional medicine throughout the world because of their effectiveness, limited side effects, and relatively low cost [5]. In particular, some phytochemicals reported to possess hypoglycemic activity have been identified in vegetal extracts such as Bitter melon, Fenugreek, Gymnema, and Morus Alba [6]. The mechanism of action of these botanicals relies on stimulation of insulin secretion and/or reduction of intestinal glucose absorption [6]. 

The plant stress hormone abscisic acid (ABA) has recently been recognized as an animal hormone involved in glycemia homeostasis [7,8]. Plasma ABA (ABAp) increases in healthy subjects after an oral glucose load and impairment of this normal ABA response to hyperglycemia occurs in T2D and in gestational diabetes (GDM) [9]. GDM is the only diabetic condition capable of spontaneous remission to apparent normalcy (after childbirth). Interestingly, the plasma ABA response to a glucose load also is restored after delivery; thus, resolution of the diabetic state after childbirth is accompanied by the restoration of a normal ABA response to oral glucose [9]. Fasting ABAp was investigated before and after biliopancreatic diversion (BPD) in obese, normal glucose tolerant (NGT) subjects, and in obese T2D patients, in which resolution of diabetes was observed after BPD. Compared to pre-BPD values, basal ABAp significantly increased 1 month after BPD in T2D as well as in NGT subjects, in parallel with a reduction of fasting plasma glucose. 

Altogether, these observations lend support to the hypothesis that endogenous (nanomolar) ABA plays a critical role in normal glucose tolerance, and that low-dose exogenous ABA may provide a means to improve glucose tolerance in prediabetes.

Impairment of the normal ABA response to hyperglycemia occurs in T2D [9], supporting the hypothesis that ABA supplementation might improve glucose tolerance in prediabetes. 

Indeed, a single ABA oral dose of 1 µg per Kg body weight (BW), in the form of an ABA-rich vegetal extract or of synthetic ABA, reduces glycemia after glucose load in healthy humans or in rodents, respectively [7]. The glycemia-lowering effect of low-dose ABA does not depend on stimulation of insulin secretion, as insulinemia is reduced in ABA-treated compared with ABA-untreated controls. The fact that low-dose ABA is not an insulin secretagogue sets ABA apart from most other current therapeutics with glycemia-lowering action, which conversely stimulate insulin release and may accelerate the beta cell demise that eventually occurs in T2D due to lifelong overstimulation.

The aim of this study was twofold: (i) to test the effect of an ABA-rich vegetal extract formulated for human use on glycemia of healthy subjects taking a single carbohydrate-rich meal or fed a Mediterranean diet for 75 days; (ii) to explore the effect of synthetic ABA on glycemia, lipidemia, and body weight in mice fed a high-glucose diet for 4 months.

## 2. Materials and Methods 

### 2.1. ABA-Containing Food Supplement

The ABA-containing food supplement was developed and notified to the Italian Ministry of Health by Nutravis S.r.l. (Genova, Italy). It contained GSECM-50^®^, a vegetal source of ABA, providing approximately 55 µg ABA/tablet.

### 2.2. Human Volunteers

Ten healthy volunteers (6 females and 4 males, aged between 31 and 58 years, mean age 43.6 years) were enrolled. They were selected among 20 prospective candidates meeting the inclusion criteria (see Table 1) because they showed values of fasting blood glucose (FBG) and/or of total cholesterol close to or slightly above the normal upper limit at the beginning of the study. All subjects gave their informed consent for inclusion before they participated in the study. The clinical trial was conducted in accordance with the Declaration of Helsinki, and the protocol was approved by the Ethical Regional Committee (ERC, Genova, Italy; prot.031REG2016, 25 July 2016).

In the first experimental protocol, each subject introduced a standardized carbohydrate-rich breakfast, one with and another without one tablet of the food supplement taken immediately before the meal. The two experiments were scheduled 1 week apart and were performed in the morning after overnight fasting. Glycemia and plasma ABA (ABAp) were measured on blood samples taken before (time zero) and 15, 30, 60, and 120 min after breakfast. Glycemia was measured with a glucometer (Bayer, Milan, Italy) and ABAp was measured by ELISA [7]. All measures were performed in duplicate.

In the second experimental protocol, the volunteers were instructed not to change their dietary habits during the study and to take one tablet of the ABA-containing food supplement daily before breakfast. At the beginning of the study (day 1) and after 75 days of treatment, a blood sample was taken from each subject after overnight fasting and waist circumference (WC) and body mass index(BMI) were measured. Values of FBG, glycated hemoglobin (HbA1c), and total and HDL cholesterol were determined by the clinical chemistry laboratory of the IRCCS San Martino in Genova.

### 2.3. Animals

Male CD1 mice (6-week old) purchased from Charles River (Milano, Italy) were housed at the animal facility of the IRCCS San Martino. All protocols of animal use were approved (authorization 349, Italian Ministry of Health, 30 August 2013). As the human prediabetic condition is believed to span a time frame of approximately 15 years, between 30 and 45 years of age, the age of the mice and the duration of treatment (4 months) were chosen in order to match the time frame at which prediabetes develops in diabetes-prone humans.

### 2.4. Animal Study

Seven-week-old mice (nine/group) fed a standard chow were administered glucose in the drinking water without (controls) or with synthetic (±)-2-cis, 4-trans abscisic acid (ABA) (Sigma Aldrich, Milano, Italy). To achieve the required daily dose of glucose (1 g/Kg BW) and of ABA (1 µg/Kg BW), the daily volume of water drank by the animals was preliminarily established. Based on this volume (5 mL/day) and taking into account an average weight of the mice of 25 g, the water administered to the animals contained 0.005 g/mL of glucose and 0.005 µg/mL of ABA. The animals were weighed weekly and the concentrations of glucose and of ABA in the water were adjusted to the mean BW in each cage. After 4 months of treatment, blood was drawn after overnight fasting to measure HbA1c (Crystal Chem Inc., Elk Grove Village, IL, USA), total cholesterol, and triglycerides (Abcam, Cambridge, UK) and body weight was measured.

### 2.5. Oral Glucose Tolerance Test (OGTT) in Mice

One week before the end of the 4-month high-glucose diet, mice were fasted for 17 h before the OGTT. Then, 1 g/Kg BW of glucose was administered by gavage in a 150-µL water solution. Blood was drawn from the tail vein before gavage (time zero) and 15, 30, 60, and 120 min after gavage. Glycemia was immediately measured with a glucometer (Bayer, Milan, Italy), and each measure was performed in duplicate.

### 2.6. Statistical Analysis

A power analysis was performed to calculate the number of participants in the human study and the number of mice in the murine study, starting from results obtained in a previous investigation with an ABA-rich vegetal extract on healthy humans and with synthetic ABA on rodents [7]. 

The normal distribution of the values obtained from the human and the murine experiments was assessed with the Vassarstats website for statistical computation [13]. Continuous variables are presented as mean ±SD. Comparisons were drawn by paired, one-tailed Student’s *t*-test, when the same direction of change was observed in all values; if not, a two-tailed *t*-test was applied. Statistical significance was always set at *p* < 0.05.

## 3. Results

### 3.1. Intake of the ABA-Rich Food Supplement Reduces the Area under the Curve (AUC) of Glycemia and Increases ABAp after a Standardized Carbohydrate-Rich Breakfast

Ten subjects (6 females and 4 males) ate a standardized carbohydrate-rich breakfast either without (B w/o fs) or with (B+fs) one tablet of the food supplement. The dose of ABA taken by the subjects was between 0.7 and 1.1 µg ABA/Kg BW considering their body weight (ranging between 51and 80 Kg). The composition of the standard meal is shown in Table 2. Carbohydrate intake was adjusted to the mean body weight difference between male (72 Kg) and female (56 Kg) participants; thus, intake of sugars was approx. 0.7g/Kg body weight in both males and females.

**Figure 1:**
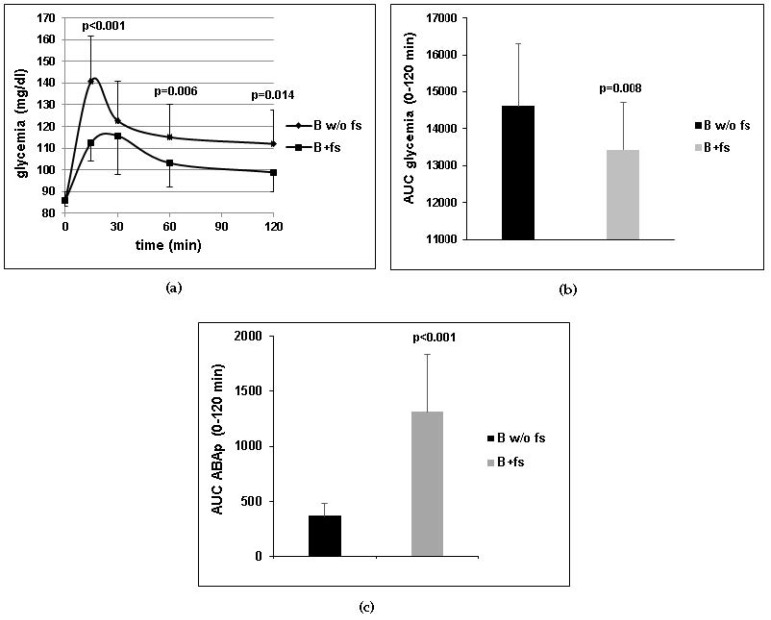
Ten healthy subjects introduced a standardized carbohydrate-rich breakfast, one with (B+fs) and another without (B w/o fs) one tablet of the food supplement, taken immediately before the meal. Mean ± SD values of (**a**) glycemia profiles; (**b**) area under the curve (AUC) of glycemia; (**c**) AUC of plasma ABA (ABAp) are shown. *p* values by paired, one-tailed *t*-test.

The endogenous ABA content of the meal (approximately 3 µg) was determined by HPLC-MS as described in [7]. Thus, the amount of ABA introduced with the B+fs was approximately 20-fold compared with the B w/o fs. Glycemia and ABAp were measured on blood samples taken immediately before (time 0) and 15, 30, 60, and 120 min after breakfast. A significant reduction of the mean glycemia profile and of the mean AUC of glycemia was observed in the B+fs experiment compared with the B w/o fs (Figure 1a,b). The mean AUC of ABAp was significantly higher in the subjects after the B+fs as compared with the B w/o fs (Figure 1c), confirming previous results indicating that oral ABA is absorbed [7]. This result indicates that intake of the ABA-rich food supplement increases plasma ABA levels and reduces glycemia in 10 subjects after a standardized carbohydrate-rich breakfast. 

### 3.2. The Daily Intake of the ABA-Containing Food Supplement for 75 Days Improves Metabolic Parameters in Healthy Subjects without Dietary Restrictions

The results obtained in the breakfast experiments prompted us to test the effect of chronic intake of the ABA-containing food supplement. One week after the end of the breakfast experiment, the same subjects were instructed to take a daily tablet of the food supplement before breakfast and not to change their feeding habits, which consisted of a typical Mediterranean diet, this being one of the inclusion criteria (Table 1B). FBG, total and HDL cholesterol, WC, and BMI were measured at days 1 and 75 of the trial. No adverse effects of the treatment were reported.

Table 3 summarizes the results obtained for each study participant. 

FBG, HbA1c, total cholesterol (TC), and BMI were all reduced at day 75 compared with day 1 in all subjects, except in subject #6, who showed a slight increase of TC, and in subjects #7 and #8, who showed very similar values for FBG and HbA1c at the start and end of the study. A significant decrease of the mean values of FBG (−20.6%), HbA1c (−5.7%), TC (−15.4%), BMI (−3.1%), WC (−5.7%), and cardiovascular risk (−17.2%) was observed after 75 days of treatment (Table 4).

The percentage of decrease was even higher when calculated only in those subjects with the highest starting value for a specific parameter (Table 5B, borderline subjects). These starting values were close to, or indeed coincided with, borderline values identified by the American Diabetes Association (ADA) guidelines for prediabetes (ADA Standard of Medical Care in Diabetes—2017) and metabolic syndrome (ATPIII Guidelines 2013): FBG ≥ 100 mg/dL; HbA1c ≥ 5.7%; TC > 200 mg/dL; non-HDL-C ≥ 160 mg/dL; WC ≥ 88 cm (females) and ≥ 102 cm (males); BMI ≥ 25 Kg/m^2^.

**Figure 2:**
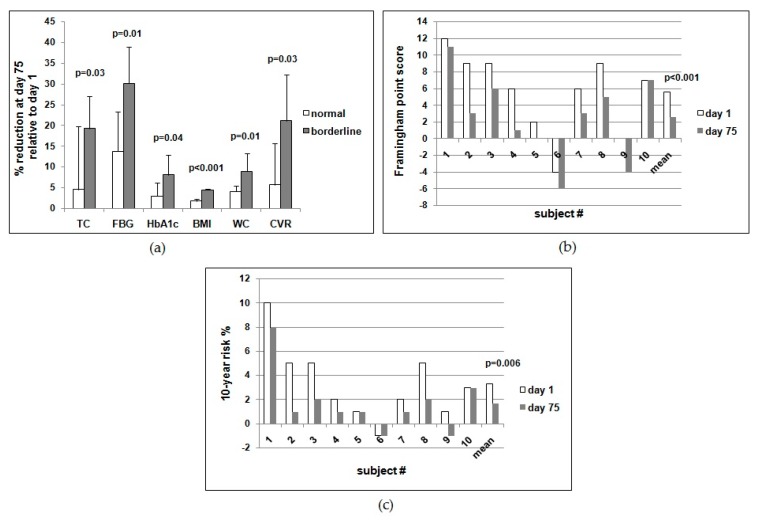
The ABA-rich food supplement taken daily for 75 days improves metabolic parameters in human subjects. In a second experimental protocol, the same volunteers who performed the breakfast experiments were instructed to take one tablet of the ABA-containing food supplement daily before breakfast for 75 days. (**a**) % of reduction at day 75 of the evaluated parameters in normal vs. borderline subjects; (**b**) Framingham point score calculated for each subject at days 1 and 75, (**c**) 10-year % risk for each subject at days 1 and 75. In panels (**b**,**c**), the last two bars are the mean of all subjects at days 1 and 75 for the considered parameter.

Thus, the mean percentages of reduction at day 75 compared with day 1 in the borderline vs. the normal subjects were 30.2 vs. 13.8 for FBG, 8.1 vs. 3.0 for HbA1c, 19.3 vs. 4.6 for TC, 26.6 vs. 12.3 for non-HDL-C, 4.5 vs. 1.8 for BMI, 9.0 vs. 4.1 for WC, and 21.2 vs. 5.7 for cardiovascular risk (Table 5). Indeed, borderline subjects improved significantly more than normal subjects at the end of the study for all parameters investigated (Figure 2a).

When the Framingham score (ATPIII Guidelines 2013) was calculated for each subject at days 1 and 75, a reduction was observed in all but one subject (#10), with a highly significant difference between the mean values at the start vs. the end of the study (Figure 2b). Accordingly, the 10-year risk percentage value was also reduced in all subjects (except for #6 and #10), with a highly significant difference between the mean values at day 1 vs. day 75 (Figure 2c).

Altogether these results indicate that the daily intake of the ABA-containing food supplement for 75 days improves markers of prediabetes (FBG and HbA1c) and metabolic syndrome (BMI, WC, TC), particularly when they are borderline.

TC, total cholesterol; FBG, fasting blood glucose; HbA1c, glycated hemoglobin; BMI, body mass index; WC, waist circumference; CVR, cardiovascular risk. P values by paired, one-tailed *t*-test.

### 3.3. Daily Intake of Synthetic ABA at 1 µg/Kg BW Improves Metabolic Parameters in Mice Fed a High-Glucose Diet

**Figure 3:**
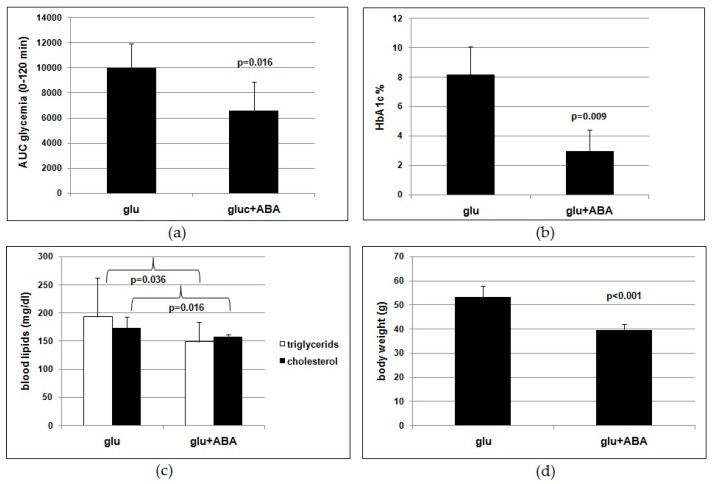
Low-dose synthetic ABA improves metabolic parameters in mice fed a high-glucose diet. Seven-week-old male CD1 mice (nine/group) were fed for 4 months a high-glucose diet containing 1 g/Kg BW glucose (administered with the drinking water) without (controls) or with ABA at approximately 1 µg/Kg BW. At the end of the study, fasting HbA1c, lipidemia, and body weight were measured and an OGTT was performed. (**a**) AUC of glycemia after OGTT, (**b**) HbA1c, (**c**) lipidemia, (**d**) body weight. Mean ± SD values are shown. *p* values by unpaired, two-tailed *t*-test.

Results obtained in the human study could have been biased by the absence of a “control” (untreated or placebo-treated group) and by the presence of other molecules besides ABA in the ABA-rich food supplement. To address these potential confounding issues, we tested the same metabolic parameters on mice fed for 4 months a high-glucose diet (1 g/Kg BW) with or without (control) 1 µg/Kg BW of synthetic ABA. An OGTT was performed after overnight fasting 1week before the end of treatment. The AUC of glycemia was significantly lower in the ABA-treated animals compared with controls (Figure 3a). At the end of treatment, HbA1c (Figure 3b), cholesterol, and triglycerides (Figure 3c) were all significantly lower in the ABA-treated animals compared with controls. Moreover, the body weight gain was reduced by 25% in the ABA-treated mice compared with controls (Figure 3d). Altogether, the results obtained indicate that chronic low-dose synthetic ABA significantly improved glucose tolerance and lipidemia in treated mice fed a high-glucose dietary regimen compared with untreated controls.

## 4. Discussion

The results of the human study indicate that a single dose of the ABA-containing food supplement ameliorated the glycemia profile of 10healthy subjects after a standardized carbohydrate-rich breakfast and that the daily intake of the food supplement for 75 days reduced FBG, HbA1c, TC, and body weight in the same subjects fed a Mediterranean diet. Approximately the same daily dose of ABA taken by each subject in the form of an ABA-containing vegetal extract (1 µg/Kg) was administered with drinking water as a synthetic molecule to mice fed a high-glucose diet for 4 months. The murine study confirms the significant reduction of glycated hemoglobin, blood lipids, and body weight in the ABA-treated group as compared with untreated controls, replicating with the pure hormone the results obtained in humans with an ABA-containing vegetal extract.

The present study originates from a previous finding that low-dose oral ABA improves glucose tolerance and reduces insulinemia in humans and rats [7,14]. The mechanism underlying the insulin-sparing effect of ABA was hypothesized to rely on stimulation by ABA of muscle glucose uptake, causing a reduction of blood glucose levels and consequently of insulin secretion. Indeed, in vitro studies have demonstrated a direct, insulin-independent effect of ABA on GLUT4 expression and membrane translocation in murine myoblasts [8]. Insulin-independent stimulation of glucose transport in the skeletal muscle may account for the glycemia-lowering effect of ABA observed both under acute (Figure 1a) and chronic (Table 4) treatment. The sparing effect of ABA on insulin release may also be partly responsible for the reduction of BMI and waist circumference observed in the clinical study (Table 3) and the reduced body weight gain observed in the ABA-treated mice fed a high-glucose diet (Figure 3d). Another possible mechanism underlying the effect of ABA on body mass is the recently reported stimulation by chronic, low-dose ABA of the expression of brown adipose tissue (BAT) marker genes in the white adipose tissue (WAT). In vitro, treatment of human and murine adipocytes with ABA induces lower triglyceride accumulation and glucose-derived fatty acid synthesis compared with insulin, increases transcription of adiponectin, and upregulates the expression of several BAT marker genes, including energy-dissipating uncoupling protein-1 (UCP1). In vivo, a single dose of ABA at 1 µg/Kg increases BAT glucose uptake twofold in rats and ABA treatment at the same dose for 1 month significantly increases expression of BAT genes in the WAT of treated mice [15]. 

Interestingly, the subjects with borderline values of FBG, TC, and/or BMI at the start of the study benefited more from the daily supplementation with ABA. As summarized in Table 5B, the reduction of FBG, TC, and HbA1c was significantly higher in the subjects with the highest levels at day 1 compared with those who had lower starting values. Indeed, borderline subjects improved significantly more compared with normal subjects for each parameter investigated (Figure 2a). This result suggests that prediabetic subjects should benefit from oral low-dose ABA supplementation, similar to the borderline subjects of this study. In this regard, it is noteworthy that the cardiovascular risk index was reduced by a significantly higher percentage in the subjects with borderline values of TC compared with those with normal starting TC values (Table 5B, Figure 2a).

Calculation of the Framingham point score for each subject at days 1 and 75 showed a reduction of the score in all subjects, except for #10, yielding a highly significant mean reduction (Figure 2b). Accordingly, the mean 10-year risk percentage value was also significantly decreased (Figure 2c).

Of note is that the significant improvement of the metabolic parameters occurred without lifestyle interventions, such as dietary restrictions or increased physical activity. One of the inclusion criteria was the dietary habit of a Mediterranean diet, defined on the basis of published criteria [10,11,12] and investigated during the interview of the candidates (Table 1). All 10volunteers were instructed not to change their dietary regimen or otherwise significantly modify their lifestyle during the study. Thus, each individual subject at the start of the study was his/her own control.

It is possible that in subjects fed a high-fat and/or high-carbohydrate diet, the effect of the ABA-containing nutraceutical could be different compared with what was observed in subjects under a Mediterranean diet. A high-fat/high-carbohydrate diet should induce faster and more pronounced changes in glucose and lipid tolerance (in diabetes-prone individuals) compared with a Mediterranean diet. Since the subjects with borderline values were those who benefited more from the nutraceutical intervention, it is possible that the beneficial effect of ABA supplementation may be more pronounced under a dietary regimen that accelerates the onset of prediabetes.

Results obtained on the murine model lend support to the conclusion that ABA is indeed the effective molecule in the food supplement. Rodents fed a high-glucose diet with synthetic ABA for 4 months show an improved glucose tolerance (Figure 3a,b) and reduced lipidemia and body weight gain (Figure 3c,d) compared with ABA-untreated controls. Thus, the outcome of the study of chronic ABA treatment in mice confirms that low-dose ABA improves the same metabolic parameters in rodents as in humans and is certainly unaffected by the presence of other vegetal-derived molecules as in the ABA-containing food supplement or by possible modifications of feeding behavior during the study.

The dose of ABA administered in the clinical and murine studies (1 µg/Kg BW) is not attainable from a vegetal-rich diet, although ABA is present in most leafy vegetables, seeds, legumes, and fruits [7,14]. Thus, intake of a food supplement containing the appropriate amount of ABA is required to achieve this dose. Absorption of ingested ABA has been already documented [7] and has been confirmed here (Figure 1c), probably occurring by simple diffusion of the protonated molecule at pH values below the pKa of ABA (4.6), such as those present in the stomach. The long half-life of ABA in the bloodstream, inferred from the elevated ABAp levels observed in humans several hours after intake of an ABA-rich extract [7], is likely due to binding of ABA to plasma proteins. Indeed, ABA binding to fatty-acid-free human albumin has been observed in vitro [7] and is reminiscent of the behavior of steroid and thyroid hormones, which share a relatively long half-life. In addition to preventing rapid urinary excretion, plasma protein binding of lipophylic hormones provides a natural slow-release reservoir, useful in lifelong treatments, where a single daily dose replenishes the bound reservoir.

## 5. Conclusions

The combined results from the human and murine studies presented here provide a strong rationale for the supplementation of human diet with microgram amounts of nutraceutical ABA as a means to correct the initial derangement of the metabolic parameters (blood glucose, lipids, and body weight) which are both symptomatic and pathogenetic of prediabetes and metabolic syndrome. Nutraceutical ABA holds promise as a simple, low-cost, and easy-to-comply-with treatment to address the urgent need for an affordable and effective means to prevent or delay the onset of T2D and cardiovascular disease. In view of the social relevance of this goal, randomized clinical studies on a higher number of prediabetic subjects are now justified to assess the beneficial potential of nutraceutical ABA, also in combination with dietary regimens different from the Mediterranean diet.

## Figures and Tables

**Table:** (**A**)

Inclusion Criteria	Exclusion Criteria
1. *Metabolic and physical parameters:* FBG ≤ 110 mg/dL; HbA1c ≤ 6.0%; TC ≤ 260 mg/dL; BMI ≤ 26 Kg/m^2^; age of ≥30 and ≤60 years. All parameters stable during the past 12 months.	1. *Metabolic parameters*: metabolic parameters as in the inclusion criteria met by medication; variation of one or more parameter by ≥10% (5% for HbA1c) of the value in the past 12 months.
2. *Lifestyle:* habitual Mediterranean diet *; sedentary to recreationally moderately active (≤2 days/week); ≤1 drink/day for women and ≤2 drinks/day for men **; non- or light-smoker (≤6 CPD).	2. *Lifestyle:* past or current self-imposed or prescribed special dietary regimes; history of eating disorders; daily or weekly physical routines of vigorous intensity.
3. *Medical examination*: in general, good health at the initial medical screening; BP ≤130/80 without anti-hypertensive medication; participation approved by the Medical Director of the study.	3. *Medical history:* past or current metabolic or endocrine disorders, cardiovascular, neurological or hematological diseases, chronic medication, history of special dietary regimes; family history of T2D and/or hyperlipidemia; planned or current pregnancy; planned surgical procedures during or immediately after the end of the study.

* As defined in refs [10,11,12]; ** Drink equivalents as defined in the 2015–2020 U.S. Dietary Guidelines for Americans; FBG = fasting blood glucose, HbA1c = glycated hemoglobin, TC = total cholesterol, BMI = body mass index, CPD= cigarettes per day, BP = blood pressure, T2D = type 2 diabetes mellitus.

**Table:** (**B**)

	Servings/Week
Vegetables	14
Fruits	21
Legumes	4
Fish	2
Nuts	3
Starches	14
White meat	≤300g
Red meat	≤100g
Olive oil as the only seasoning and cooking ingredient	

**Table:** (**C**)

Age	Mean: 43.6; median: 44.5	
	Mean age females: 46	
	Mean age males: 40	
	0–29	0
	30–39	2 (20%)
	40-49	6 (60%)
	50–59	2 (20%)
	≥60	0
Gender	4 males (40%) and 6 females (60%)	
Ethnicity	All participants were Caucasian	
Educational levels	University degree 9/10 (90%)	
	Diploma 1/10 (10%)	
Marital status	Married 4/10 (40%)	
	Single 6/10 (60%)	

**Table 2:** Composition of the standardized carbohydrate-rich breakfast.

	Males				Females	
	g or mL	Carbohydrates (g/100g)	of which Sugars (g/100g)	g or mL	Carbohydrates (g/100g)	of which Sugars (g/100g)
Biscuits	117	79.2	22.5	78	52.8	15.0
Jam	50	29.0	25.0	50	29.0	25.0
Cornflakes	25	21.7	9.2	25	21.7	9.2
Milk	150	7.8	7.8	150	7.8	7.8
**Total**		**137.7**	**64.5**		**111.4**	**57.0**
Abscisic acid (ABA) content		3.1 µg			2.9 µg	

**Table 3:** Individual metabolic and body mass parameters of 10subjects treated for 75 days with the ABA-rich food supplement.

		Age	FBG (mg/dL)	HbA1c (%)	TC (mg/dL)	HDL (mg/dL)	non-HDL-C (mg/dL)	CVR	BMI (Kg/m^2^)	WC (cm)
#1	F	58	106 vs. 73	5.9 vs. 5.1	257 vs. 214	80 vs. 77	177 vs. 137	3.2 vs. 2.8	23.0 vs. 22.5	83 vs. 78
#2	M	49	92 vs. 84	4.9 vs. 4.7	222 vs. 149	49 vs. 56	173 vs. 93	4.5 vs. 2.7	25.5 vs. 24.3	105 vs. 95
#3	F	45	92 vs. 75	5.4 vs. 5.2	210 vs. 167	56 vs. 50	154 vs. 117	3.8 vs. 3.3	21.1 vs. 20.7	84 vs. 80
#4	M	40	98 vs. 57	5.3 vs. 5.0	207 vs. 157	48 vs. 45	159 vs. 112	4.3 vs. 3.5	21.3 vs. 20.8	90 vs. 86
#5	F	44	89 vs. 79	5.6 vs. 5.3	185 vs. 148	69 vs. 54	116 vs. 94	2.7 vs. 2.7	25.8 vs. 24.6	93 vs. 81
#6	M	30	86 vs. 66	5.1 vs. 4.9	171 vs. 189	48 vs. 60	123 vs. 129	3.6 vs. 3.1	22.8 vs. 22.5	89 vs. 87
#7	M	41	75 vs. 76	5.6 vs. 5.3	204 vs. 180	46 vs. 53	158 vs. 127	4.4 vs. 3.4	22.8 vs. 22.4	87 vs. 84
#8	F	47	93 vs. 72	5.0 vs. 5.1	211 vs. 191	54 vs. 62	157 vs. 129	3.9 vs. 3.1	24.2 vs. 23.2	81 vs. 77
#9	F	31	97 vs. 76	5.2 vs. 5.0	211 vs. 170	103 vs. 98	108 vs. 72	2.0 vs. 1.7	21.0 vs. 20.8	72 vs. 70
#10	F	51	96 vs. 71	5.0 vs. 4.8	190 vs. 182	78 vs. 72	112 vs. 110	2.4 vs. 2.5	21.0 vs. 20.6	83 vs. 79

Values recorded at days 1 and 75 of the study are shown for each parameter. FBG, fasting blood glucose; HbA1c, glycated hemoglobin; TC, total cholesterol; non-HDL-C, non-HDL cholesterol, calculated as the difference between TC and HDL; CVR, cardiovascular risk, calculated as the ratio between TC and HDL; WC, waist circumference. “Borderline” values are shaded in grey (see Results).

**Table 4:** Pre- vs. post-treatment comparison of the metabolic parameters explored in 10subjects treated for 75 days with the ABA-rich food supplement.

Parameter	Day 1	Day 75	% Reduction	*p* Value
FBG (mg/dL)	92 ± 8.2	73 ± 7.4	20.6	<0.001
HbA1c (%)	5.3 ± 0.3	5.0 ± 0.2	5.7	0.0066
Total cholesterol (mg/dL)	207 ± 23	175 ± 20	15.4	0.0014
BMI (Kg/m^2^)	22.9 ± 1.8	22.2 ± 1.5	3.1	<0.001
Waist circumference (cm)	87 ± 8.7	82 ± 7.0	5.7	<0.001
Cardiovascular risk	3.49 ± 0.9	2.89 ± 0.5	17.2	0.0048

Mean values± SD of each parameter were calculated on all study participants (see Table 3). Day 1= start of the study; day 75= end of the study. The cardiovascular risk was calculated for each subject as the ratio between total cholesterol and HDL. *p* value (day 1 vs. day 75) by paired, one-tailed *t* -test.

**Table:** (**A**)

Parameter	Normal Subjects (#)	Day 1	Day 75	% Reduction	*p* Value
FBG (mg/dL)	2; 3; 5; 6; 7; 8	88 ± 6.8	75 ± 6.1	13.8 ± 9.5	0.008
HbA1c (%)	2; 3; 4; 6; 8; 9; 10	5.1 ± 0.2	5.0 ± 0.2	3.0 ± 3.2	0.021
TC (mg/dL)	5; 6; 10	182 ± 9.8	173 ± 21.9	4.6 ± 15.3	n.s.
Non-HDL-C (mg/dL)	5; 6; 9; 10	115 ± 6	101 ± 24	12.3 ± 17.2	n.s.
BMI (Kg/m^2^)	1; 3;4; 6; 7; 9; 10	21.9 ± 1.0	21.5 ± 0.9	1.8 ± 0.4	<0.001
Waist circumference (cm)	1; 3; 6; 7; 8; 9; 10	83 ± 5.4	79 ± 5.4	4.1 ± 1.3	<0.001
Cardiovascular risk	1; 5; 9; 10	2.6 ± 0.5	2.4 ± 0.5	5.7 ± 10.1	n.s.

**Table:** (**B**)

Parameter	Borderline Subjects (#)	Day 1	Day 75	% Reduction	*p* Value
FBG (mg/dL)	1; 4; 9; 10	99 ± 5.7	69 ± 11.3	30.2 ± 8.7	0.003
HbA1c (%)	1; 5; 7	5.7 ± 0.2	5.2 ± 0.2	8.1 ± 4.7	0.050
TC (mg/dL)	1; 2; 3; 4; 7; 8; 9	217 ± 18.3	175 ± 21	19.3 ± 7.8	<0.001
Non-HDL-C (mg/dL)	1; 2; 3; 4; 7; 8	163 ± 10	119 ± 16	26.6 ± 10.4	0.001
BMI (Kg/m^2^)	2; 5; 8	25.2 ± 0.9	24.0 ± 0.7	4.5 ± 0.3	0.002
Waist circumference (cm)	2; 4; 5	96.0 ± 7.9	87.0 ± 7.1	9.0 ± 4.2	0.034
Cardiovascular risk	2; 3; 4;6; 7; 8	4.1 ± 0.4	3.2 ± 0.3	21.2 ± 11.0	0.005

Mean ± SD values of each parameter were calculated separately in “normal” (panel A) subjects (unshaded in Table 3) and in borderline subjects (panel B), i.e., those with starting values of FBG ≥ 96 mg/dL, HbA1c ≥ 5.6%, TC > 200 mg/dL, non-HDL-C ≥ 154 mg/dL; BMI > 24 Kg/m^2^, waist circumference ≥ 90 cm (grey-shaded in Table 3). *p* by paired, one-tailed *t-*test; n.s., not significant. FBG, fasting blood glycemia; HbA1c, glycated hemoglobin; TC, total cholesterol.
